# Amorphous Drug-Polymer Salts

**DOI:** 10.3390/pharmaceutics13081271

**Published:** 2021-08-17

**Authors:** Xin Yao, Amy Lan Neusaenger, Lian Yu

**Affiliations:** School of Pharmacy and Department of Chemistry, University of Wisconsin-Madison, Madison, WI 53705, USA; xyao39@wisc.edu (X.Y.); neusaenger@wisc.edu (A.L.N.)

**Keywords:** amorphous, crystallization, tropical conditions, global health, polyelectrolytes, coating, drug–polymer salt

## Abstract

Amorphous formulations provide a general approach to improving the solubility and bioavailability of drugs. Amorphous medicines for global health should resist crystallization under the stressful tropical conditions (high temperature and humidity) and often require high drug loading. We discuss the recent progress in employing drug–polymer salts to meet these goals. Through local salt formation, an ultra-thin polyelectrolyte coating can form on the surface of amorphous drugs, immobilizing interfacial molecules and inhibiting fast crystal growth at the surface. The coated particles show improved wetting and dissolution. By forming an amorphous drug–polymer salt throughout the bulk, stability can be vastly enhanced against crystallization under tropical conditions without sacrificing the dissolution rate. Examples of these approaches are given, along with suggestions for future work.

## 1. Introduction

An amorphous drug has a higher solubility than its crystalline counterpart, providing a general approach to improving the solubility and bioavailability of drugs [1,2,3]. Drugs considered for amorphous formulations are often hydrophobic and poorly water soluble, belonging to Class II and IV of the Biopharmaceuticals Classification System. These drugs are often dispersed in hydrophilic polymers, producing the so-called amorphous solid dispersions (ASDs), to help their dispersion and dissolution in water.

Medicines for global health should be stable under the highly stressful tropical conditions (high temperature and high humidity) and often require high drug loading. These requirements present additional challenges for amorphous formulations. Moisture is a potent mobility-enhancer and can dramatically accelerate the crystallization of amorphous drugs [4,5], especially when combined with high temperature. For this reason, the combination of 40 °C and 75% RH (the “G condition”) is the harshest for pharmaceutical stability testing and the highest bar for the stability of amorphous drugs. High drug loading is desirable for those global-health medicines that have a high pill burden; for example, treatment for HIV requires 3–12 pills per day [6,7]. High drug loading in a single dosage form reduces patient discomfort and improves compliance.

Here, we discuss our recent work performed with support from the Gates Foundation to develop stable amorphous formulations for global health. Based on the needs for global health discussed above, our goal is a low-cost manufacturing strategy for amorphous formulations with high drug loading and high stability under tropical conditions. We show that amorphous drug–polymer salts offer a promising approach toward this goal. This strategy can be implemented by (1) local salt formation (polyelectrolyte coating) on the surface of an amorphous drug and (2) uniform salt formation throughout the bulk. We provide examples that illustrate this strategy and the improvement of formulation performance as a result.

A drug–polymer salt is produced by an acid-base reaction between a small-molecule drug and an ionizable polymer (polyelectrolyte). Although salt formation is a common approach in drug development [8,9,10,11], the counterions are typically small inorganic ions or small organic ions, not charged polymers. We will demonstrate the special advantages of polymeric counterions in salt formation. In the context of amorphous formulations, a salt formed with a polymeric counterion has greater resistance to crystallization than a salt formed with a small inorganic or organic counterion. This is a result of the awkward packing required for a drug and a polymer to crystallize together. Additionally, polyelectrolytes tend to be hydrophilic, and their incorporation into a drug formulation improves wetting and dispersion in water. A polymer has a lower solubility than its monomer or oligomers and provides stronger adhesion to solid surfaces. As a result, polyelectrolytes are often good coating materials, while low-molecular-weight materials could fail for this purpose.

A polyelectrolyte is a polymer in which all (or nearly all) monomer units can be ionized depending on pH. Polyelectrolytes are useful as viscosity enhancers (thickeners), emulsifiers, modifiers/stabilizers of colloidal structures, and coating materials. They have many applications in a vast array of industries. Many polyelectrolytes are acceptable ingredients in food and drugs; for example, pectin as a thickener and gelling agent in jams and jellies [12]. Polyelectrolytes are used to stabilize nanoparticle suspensions [13]. They play an important role in the formulation of hydrogels with unique characteristics such as self-healing and viscoelasticity (important for bioplastics) [14] and responsiveness to external stimuli (useful for sensors and drug delivery vehicles) [15,16,17,18]. A key application of polyelectrolytes for this work is their ability to produce ultra-thin coatings through electrostatic deposition and layer-by-layer assembly [19]. The ultra-thin coatings have been used to control drug release [20], stabilize drug delivery vehicles [21], and protect medical devices from causing fungal infections [22]. This work is concerned with the applications of polyelectrolytes in stabilizing amorphous drugs.

**Figure 1:**
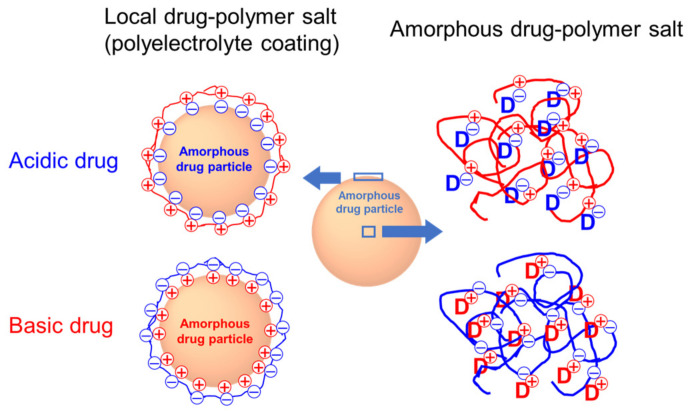
The two types of salt formation between drugs and polyelectrolytes investigated in this work. Left: Local salt formation (polyelectrolytes coating) on the surface of amorphous particles. Right: Uniform amorphous drug–polymer salt throughout the bulk. Each sphere represents an amorphous solid particle. “D” designates a drug molecule.

As illustrated in Figure 1, we investigate two modes of salt formation between an amorphous drug and a polymer. Polyelectrolytes are used to form ultra-thin coatings on the surface of amorphous drugs (left). An acidic drug exposes negative charges in an aqueous medium (with pH > pK_a_) and can be coated with polycations. Likewise, a basic drug exposes positive charges in an aqueous medium (with pH < pK_a_) and can be coated with polyanions. Due to charge neutralization, a polyelectrolyte coating is extremely thin, approximately a monolayer, a property useful for achieving a high drug loading. On the right of Figure 1, we show similar processes of salt formation but shift our focus to the bulk material. By reacting acidic (basic) drugs with basic (acidic) polymers, amorphous salts can be formed throughout the materials, not just on the surface. These two modes of salt formation will be discussed in Section 2 and Section 3.

## 2. Polyelectrolyte Coating

**Figure 2:**
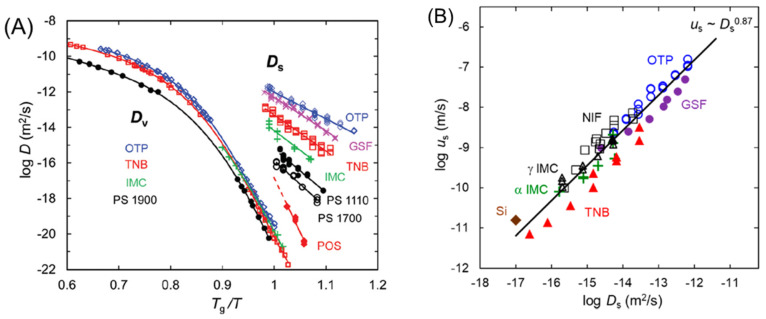
(**A**) Diffusion coefficient on the surface (*D*_s_) and in the bulk (*D*_v_) of several glass-forming molecular liquids against a *T*_g_-scaled temperature [33]. Reproduced with permission from [33], Royal Society of Chemistry, 2020. (**B**) Crystal growth rate on the surface *u*_s_ plotted against the surface diffusion coefficient *D*_s_ for molecular glasses and amorphous silicon. OTP: ortho-terphenyl. GSF: griseofulvin. TNB: tris-naphthyl benzene. NIF: nifedipine. IMC: indomethacin. POS: posaconazole. PS: polystyrene oligomers [35]. Reproduced with permission from [35], American Chemical Society, 2017.

Recent work has shown that amorphous drugs can grow crystals much faster at the free surface than in the bulk [23,24,25,26,27,28]. This is a result of the high mobility of molecules on the surface [29,30,31,32,33] and has motivated the development of surface coatings to stabilize amorphous drugs. Figure 2A shows the diffusion constants measured on the surface and in the interior of molecular glasses, many of which are amorphous drugs. Surface diffusion can be much faster than bulk diffusion by up to eight orders of magnitude when compared at the glass transition temperature *T*_g_, and the difference increases with cooling [34,35]. High surface mobility is a result of fewer neighbors surrounding a surface molecule relative to a bulk molecule, making it freer to move. The greater variation of surface mobility relative to bulk mobility is a consequence of the different degrees to which surface molecules are liberated relative to bulk molecules.

Fast surface diffusion leads to fast surface crystal growth. In Figure 2B, the surface crystal growth rate *u*_s_ is plotted against the surface diffusion coefficient *D*_s_, and we observe a nearly proportional relation, *u*_s_ ~ *D*_s_^0.87^. That is, the faster the surface diffusion, the faster the surface crystal growth by about the same factor. This supports the notion that surface crystal growth is controlled by surface diffusion [34,35]. This conclusion is further supported by the fact that surface crystals grow upward and laterally without deep penetration into the bulk and are surrounded by grooves created by the surface motion of molecules toward the crystal [36,37].

Fast surface crystallization presents a significant threat to the stability of amorphous drugs. All amorphous formulations have free surfaces and internal voids where crystallization can be accelerated by enhanced surface mobility. The problem worsens for formulations with high surface-to-volume ratios, including powders, thin films, and nanoparticles [23]. However, since surface crystallization is highly localized, the problem can be solved with a local solution—surface coatings. A coating, in essence, converts surface molecules into bulk molecules, thus eliminating surface crystallization. As we discuss below, surface coating by polyelectrolytes provides an ultra-thin nano-coating with many advantages: stability against crystallization, high drug loading, improved wetting, fast dissolution, good powder flow, and direct tabletability. Table 1 shows examples of the polyelectrolyte coating of amorphous drugs. Specific examples are discussed below.

**Figure 3:**
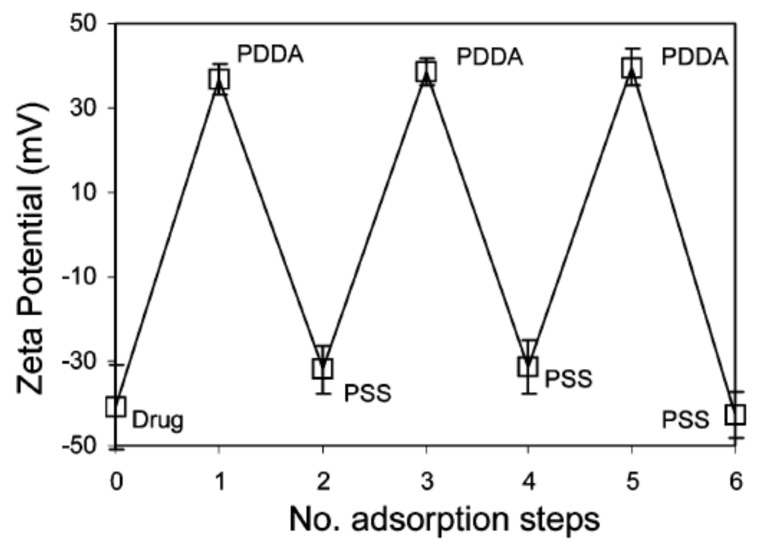
Zeta potential of amorphous IMC particles versus the number of adsorption steps [38]. Reproduced with permission from [38], American Chemical Society, 2007.

Wu et al. first demonstrated the use of a polyelectrolyte coating to inhibit surface crystal growth on an amorphous drug [38]. They coated amorphous indomethacin (IMC), a weak acid with p*K*_a_ = 4.5, with the polycation PDDA (polydiallyldimethylammonium) in an aqueous solution. At the coating pH (6.1), the IMC and PDDA are oppositely charged, allowing for electrostatic deposition and the reversal of surface charge (Figure 3). In contrast, the polyanion PSS (poly(styrenesulfonate)) cannot directly coat IMC due to charge repulsion, but it can deposit on a previously coated layer of PDDA. A multilayer coat can be produced by alternate deposition of PDDA and PSS. Wu et al. found that coated amorphous IMC is significantly more stable against crystallization than uncoated amorphous IMC. The effect is pronounced even with a single coat of PDDA: after 20 days at 40 °C, an uncoated sample is fully covered by crystals, while a coated sample has a coverage of only several percent. This ultra-thin coating helps achieve a very high drug loading and improves the flowability of drug powders (the angle of repose is reduced from 36° to 18°).

**Figure 4:**
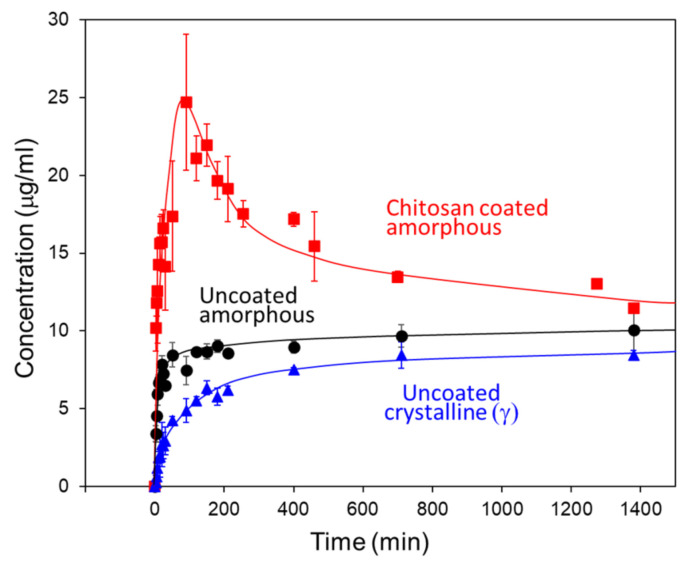
The effect of chitosan coating on the dissolution rate of amorphous IMC particles at 37 °C [41]. Reproduced with permission from [41], American Chemical Society, 2019.

Further work on surface coating employed amorphous IMC as a model substrate and pharmaceutically acceptable polymers as coating materials (PDDA is not a pharmaceutical excipient). For example, Li et al. performed a comprehensive study of the effect of chitosan coating on the properties of amorphous IMC [41]. Chitosan is a natural basic polymer (p*K*_a_ = 6.5). Though weaker than PDDA as a polyelectrolyte, a chitosan coating similarly eliminates surface crystallization in IMC for samples tested in both film and particle geometries. Li et al. also compared chitosan with gelatin, an even weaker polyelectrolyte, as coating materials and found chitosan-coated particles to be more stable against crystallization and to remain free-flowing upon storage, whereas gelatin-coated particles became sticky after storage at high humidity and clumped together. Importantly, chitosan-coated amorphous particles dissolved in water faster than uncoated particles (Figure 4) [41]. The improvement is a result of better wetting and the slower crystallization of coated particles during dissolution. These effects apparently outweigh the barrier effect of the polymer coating in drug release. The thin chitosan coating also improved powder flow and tabletability.

Subsequent work on polyelectrolyte coating extended beyond the acidic drug IMC to include basic drugs (Table 1). These studies applied the same principle of coating illustrated in Figure 1 but used polyanions to coat the positively charged surfaces of basic drugs. For the basic drug clofazimine (CFZ, p*K*_a_ = 8.5), Gui et al. investigated the coating of alginic acid (p*K*_a_ = 3.5) [42]. They performed the coating in an aqueous solution at pH 7 so that the drug and the polymer were oppositely charged to allow for electrostatic deposition. The coating effect on stability was evaluated for particles stored at 90 °C and 40 °C/75% RH. At 90 °C, the coated particles did not crystalize in 60 days, while the uncoated particles fully crystallized. At 40 °C/75% RH, the coated particles crystallized approximately three times slower than the uncoated particles. The coated particles dissolved faster in Simulated Gastric Fluid (SGF) than the uncoated particles and showed more prolonged supersaturation (the “spring-and-parachute” profile). Within one hour, the coated particles dissolved two times faster than the uncoated amorphous particles and three times faster than the uncoated crystalline particles. As in the case of chitosan-coated IMC, the alginate coating improved the wetting of the coated particles and slowed their crystallization during dissolution.

Besides polyelectrolyte coatings, other coating methods have been used to improve the properties of amorphous formulations, both solvent-based [44,45,46,47,48] and solvent-free [39,49,50,51,52]. Relative to the other methods, polyelectrolyte coatings applied via electrostatic deposition are characterized by extremely small thickness (several to tens of nanometers per layer). Even at this thickness, the coating eliminates surface crystallization. This coating method differs from many others in that an aqueous coating solution is used. An aqueous medium is compatible with poorly water-soluble drugs, in which they are present as undissolved, solid particles to be coated, while the use of organic solvents may dissolve the drugs. Polyelectrolyte coating is applied using a simple dip-coating process, which ensures coating uniformity. Owing to the small amount of coating material in the final product, this method helps achieve high drug loading while saving room in the formulation for other functional excipients. Enumerating these advantages is not to imply that polyelectrolyte coating is superior to other coating methods in all respects. A thicker coating is required for applications where solid particles collide, causing coatings to wear off, and where a thicker layer is needed for the passage through the stomach for controlled release.

## 3. Amorphous Drug–Polymer Salts in the Bulk

Although a thin surface coating can eliminate surface crystallization, many amorphous drugs crystallize so rapidly in the bulk (especially under the stressful tropical conditions) that additional protection is needed. Furthermore, what appears to be a contiguous bulk material may, in fact, contain voids and fractures that lead to fast local crystallization [53]. This internal process can propagate in a vicious cycle through additional fracture and additional crystal growth [53]. There has been extensive work on the use of polymers as inhibitors of bulk crystallization [54,55,56]. The ensuing discussion focuses on the use of drug–polymer salts to stabilize amorphous formulations under what is perhaps the harshest condition for stability testing, 40 °C/75% RH, without sacrificing dissolution performance. This condition presents an ultimate separator for stabilization strategies. For example, surface-coated amorphous IMC is quite stable at 40 °C and in low humidity but quickly crystallizes at 40 °C/75% RH [41], indicating the need for further stabilization.

In Table 2, we summarize the examples of amorphous drug–polymer salts with attention to synthetic methods, drug loading, stability at 40 °C/75% RH, and dissolution performance. This is followed by case studies and general comments.

Given the difficulty of processing high polymers, the method of forming drug–polymer salts deserves some discussion. According to the literature, drug–polymer salts can be prepared using many methods, including hot-melt extrusion (HME), ball milling, cryogenic milling, solvent evaporation such as spray- and freeze-drying, mixing solutions, and slurry conversion. The first two methods require no solvents. HME achieves the uniform mixing of components by heat, pressure, and physical mixing [68]. In ball milling and cryogenic grinding, solid components are mixed along with particle size reduction [69]. The other methods on the list above require the use of solvents, which help lower the processing temperature (necessary for thermally labile drugs and polymers) and increase the rate of mass transport [70]. The solvent evaporation method requires a common solvent for the drug and the polymer, which could be difficult to find when the polymer is hydrophilic (an electrolyte) and the drug is hydrophobic and poorly water soluble. The mixing of two solutions, one of the drug and the other of the polymer, has been used to prepare drug–polymer salts (“complexes”) and nanoparticles [64]. In our work, slurry conversion was used as a low-cost method to prepare amorphous drug–polymer salts [64]. In this method, solid components are mixed in the presence of a small amount of solvent with mild heating and stirring. Since equilibration is slow in a polymer system, there is room for future optimization and innovation in engineering the structures of amorphous drug–polymer salts.

**Figure 5:**
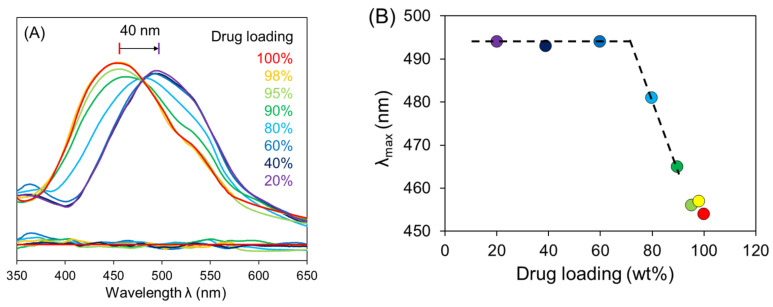
(**A**) Visible absorption spectra of amorphous CFZ−PAA films at different drug loading. (**B**) λ_max_ (wavelength of maximal absorption) vs. drug loading. The same color coding is used in (**A**,**B**). By extrapolation, the saturation drug loading is determined at 70% [64]. Reproduced with permission from [64], American Chemical Society, 2021.

We illustrate the formation of amorphous drug–polymer salts and their pharmaceutical benefits using the reaction of the acidic polymer poly(acrylic acid) (PAA) with two basic drugs, clofazimine (CFZ) [64] and lumefantrine (LMF) [65]. For both systems, a simple slurry method was used to produce the amorphous salt at a high drug loading (75% for CFZ–PAA and 50% for LMF–PAA). The synthesis was performed under a mild condition suitable for thermally unstable drugs and polymers. The salt formation was confirmed by spectroscopy, and we illustrate this for CFZ–PAA (Figure 5) [64]. With increasing drug loading, the visible absorption spectrum initially does not change much but then undergoes a blue shift, eventually becoming the spectrum of the free base. The evolution is well fitted by a two-state model and exhibits an isosbestic point, indicating an equilibrium between the neutral and the ionized drug molecules. The spectral shift indicates a saturation drug loading of 70%, above which the drug–polymer mixture contains neutral drug molecules. For both CFZ and LMF, the salt formation with PAA elevates the glass transition temperature *T*_g_ above the *T*_g_ of the polymer (126 °C), indicating significant reduction of molecular mobility.

**Figure 6:**
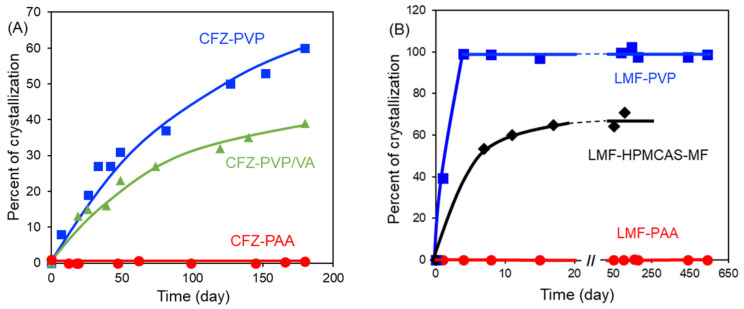
Stability of amorphous drug–polymer salts at 40 °C/75% RH. (**A**) CFZ−PAA (75% drug loading) [64]. No crystallization was observed in 6 months, while the neutral CFZ−PVP and CFZ−PVP/VA dispersions at the same drug loading both crystallized. Reproduced with permission from [64], American Chemical Society, 2021. (**B**) LMF–PAA salt (50% drug loading) [65]. No crystallization was observed after 18 months for LMF–PAA, while the neutral LMF−PVP and partially ionized LMF−HPMCAS dispersions at the same drug loading both crystallized. Reproduced with permission from [65], Elsevier, 2021.

For both CFZ and LMF, salt formation vastly improves the stability against crystallization at 40 °C/75% RH (Figure 6). No crystallization was observed in CFZ–PAA at 75% drug loading for at least 6 months, while the neutral dispersion of unionized CFZ in PVP or PVP/VA began crystallizing within weeks. In the case of the amorphous LMF–PAA salt (50% drug loading), no crystallization was observed for at least 18 months, while the neutral dispersion in PVP or HPMCAS began to crystallize within weeks. Despite the higher stability, these amorphous drug–polymer salts showed fast dissolution and extended supersaturation in biorelevant media SGF and FaSSIF [64,65]. Their solid particles remained free flowing after storage at 40 °C/75% RH and showed improved tabletability.

When a comparison is possible, polymers that allow for salt formation with the drug appear to inhibit crystallization better than those that do not. Some of these cases are given in Table 2. For example, indomethacin is more stable when formulated with Eudragit EPO, a salt former, than with HPMC, a neutral, non-salt-forming polymer [60]. Clofazimine and lumefantrine, both bases, are more stable when formulated with an acidic polymer than with a neutral polymer [62,64,65]. This confirms the importance of salt formation on the stability of amorphous drug–polymer formulations.

**Figure 7:**
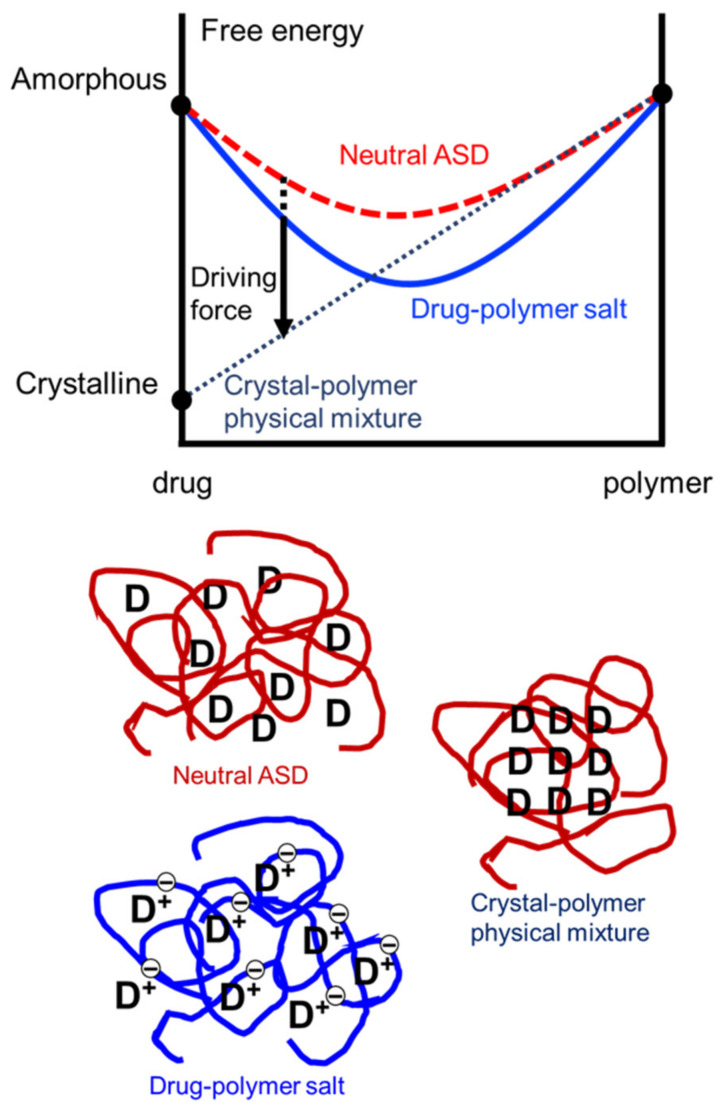
Free-energy diagram for crystallization in an amorphous salt and a neutral ASD. A drug–polymer salt has lower free energy than a neutral ASD because of the strong ionic interactions, leading to a lower driving force for crystallization. The drug is assumed to be a base. The drawings on the bottom represent a neutral ASD, a drug–polymer salt, and the crystallized drug in a polymer matrix [65]. Reproduced with permission from [65], Elsevier, 2021.

Why is an amorphous drug–polymer salt so stable against crystallization at high temperatures and in high humidity? Crystallization requires a driving force and molecular mobility. The formation of a drug–polymer salt simultaneously reduces the driving force and molecular mobility. Because of strong ionic interactions, salt formation reduces the system’s free energy to a greater extent than the mixing of neutral components. This is illustrated in Figure 7. The large free energy of mixing leads to a lower (even zero or negative) driving force for crystallization. In Figure 7, we imagine a drug dissolved in a non-crystallizing polymer such as PVP and PAA, for which the only practical pathway of crystallization is the formation of drug crystals. This is because it is nearly impossible for the drug and the polymer to crystallize together in the same unit cell.

The low molecular mobility of an amorphous salt is a consequence of its high *T*_g_. Salt formation is observed to elevate the *T*_g_ to a greater extent than the mixing of neutral components [64,65]. Given that amorphous systems have similar mobility at *T*_g_, this means that an amorphous salt has substantially lower mobility than a neutral dispersion when stored at the same temperature. Our discussion above indicates that by forming a salt with a polymer, a drug has a lower driving force to crystallize, as well as lower mobility available for crystallization. This leads to high stability against crystallization even under the highly stressful tropical conditions.

## 4. Concluding Remarks

In this perspective, we discussed the role of drug–polymer salts in stabilizing amorphous drug formulations and improving other pharmaceutical properties. Through local salt formation, an ultra-thin layer of polyelectrolyte can be coated on the surface of amorphous drugs. The thin coating inhibits surface crystallization with a minute amount of coating material and improves wetting, dissolution, power flow, and tableting. With uniform salt formation throughout the bulk, stability against crystallization can be vastly improved under the harshest condition for stability testing, 40 °C/75% RH, without sacrificing the dissolution rate. This effect arises because of the difficulty or inability for the drug and the polymer to crystallize together, the significantly reduced driving force for crystallization, and the increased kinetic barrier for molecular motions. Despite their greater stability, amorphous drug–polymer salts can dissolve rapidly.

One possible area for future work is the optimization of the salt-forming process. The low mobility of high polymers makes the state of a drug–polymer mixture not only a matter of thermodynamics (the tendency for mixing) but also a matter of kinetics (the rate of mixing). To illustrate the kinetic control in this context, consider the different manners in which a small amount (~1%) of the acidic polymer PAA can be incorporated into the basic drug clofazimine: depending on the processing conditions, PAA can be introduced as a surface coating or a bulk additive, both products being kinetically stable [64]. Such flexibility would be difficult to achieve with a small-molecule second component. At present, there are many methods for forming drug–polymer salts, both solvent-free and solvent-assisted. It is of interest to characterize the microstructures of these products for the uniformity and degree of ionization. One parameter to be optimized is the molecular weight of the polyelectrolyte for salt formation. A higher molecular weight could mean a higher *T*_g_ and better stability of the amorphous salt, but it might also lead to low solubility, slow drug release, and high viscosity of manufacturing solutions [71,72]. Another parameter to be optimized is the drug–polymer ratio. For clofazimine–PAA (Figure 6), 70% is the maximal drug loading that ensures full ionization. It is of interest to learn whether this should be viewed as the upper limit for drug loading or if even higher loading should be attempted, yielding a mixture of salt and free base without sacrificing stability. These formulation parameters will need to be weighed against their impact on product performance, including stability and dissolution.

Another area of potential future work is the application of polyelectrolyte chemistry to improve drug delivery. Polyelectrolytes have been used to stabilize nanoparticle suspensions [13] and form hydrogels [14]. This property could be related to the observation of colloidal particles during the dissolution of amorphous drug–polymer salts [62,65]. It is of interest to learn whether this is a general property of drug–polymer salts and, if so, whether it has any pharmaceutical applications. An interesting property of drug–polymer salts is that drug release can be triggered by the increase in ionic strength [67]. This property could be useful for the controlled release of drugs.

## Figures and Tables

**Table 1:** Examples of polyelectrolyte-coated amorphous drugs.

Drug	Polymer	Stability against Crystallization	Other Benefits	Reference
*Acids*				
Indomethacin	PDDA	Stable at 40 °C/dry for 20 d, while uncoated sample fully crystallized	Improved flowability	[38]
Indomethacin	Eudragit EPO (dry coating)	Improved stability at 30 °C/23% or 42% RH, outperforming neutral polymer Soluplus	No tests performed	[39]
Indomethacin	Gelatin A and B	Inhibited surface crystal growth at 40 °C/dry	No tests performed	[40]
Indomethacin	Chitosan, gelatin A and B	Improved stability at 40 °C/dry, 40 °C/75% RH, and 30 °C/75% RH; chitosan outperformed gelatins	Improved powder flow, tabletability, and wetting and dissolution	[41]
*Bases*				
Clofazimine	Alginic acid	Stable at 90 °C/dry for 60 d, while the uncoated particles fully crystallized. Improved stability at 40 °C/75% RH	Improved wetting and dissolution	[42]
Nifedipine	Gelatin A and B	Inhibited surface crystal growth at 40 °C/dry	No tests performed	[40]
Loratadine	Dextran sulfate (DTS)	Improved stability at 40 °C/dry	No tests performed	[43]

**Table 2:** Examples of amorphous drug–polymer salts.

Drug, % Loading	Polymer	Synthesis Method	Physical Stability	Other Benefits	Reference
*Acids*					
Naproxen, 42%	Eudragit EPO	Hot melt extrusion	Stable at 20 °C/60% RH for 12 mo.	Drug release triggered by inorganic salts	[57]
Mefenamic acid, 24%	Eudragit EPO, Eudragit L100	Cryogenic grinding	Stable at 25 °C/75% RH for 10 mo.	Extended supersaturation, enhanced dissolution	[58]
Lapatinib, 40% Gefitinib, 40%	PSSA	Solvent evaporation, cryogenic grinding	Stable at 40 °C/75% RH for 6 mo.	Faster dissolution than crystalline form	[59]
Indomethacin, 30%	Eudragit EPO	Solvent evaporation, cryogenic grinding	Stable at 40 °C/75% RH for 100 d. Neutral ADSs less stable	Enhanced dissolution	[60]
*Bases*					
Pyrimethamine, Lamotrigine, Trimethoprim, <65%	Polyacrylic acid (PAA)	Melt quench	Stable at 40 °C/75% RH for 6 mo. Pure drugs, neutral ASDs less stable	Fast dissolution relative to the crystalline and persisting supersaturation	[61]
Lumefantrine, 40%	CAP, HPMCP, Eudragit L100	Solvent evaporation	Stable at 40 °C/75% RH for 6 mo. Neutral ASDs less stable	CAP dispersion shows slow dissolution; others perform better	[62]
Clofazimine, 33–57%	HPMCP	Solvent evaporation	Not performed	Not performed	[63]
Clofazimine, 75%	PAA	Slurry conversion	Stable at 40 °C/75% RH for 6 mo. Neutral ASDs less stable	Improved flow, tabletability, wetting, and dissolution	[64]
Lumefantrine, 50%	PAA	Slurry conversion	Stable at 40 °C/75% RH for 18 mo. Neutral ASDs less stable	Improved flow, tabletability, and dissolution	[65]
Ciprofloxacin, 40%	Eudragit L	Ball milling	Stable at 25 °C/90% RH for 90 min. Improved stability over pure drug at 40 °C/75% RH	Improved solubility and drug permeability, persistent supersaturation	[66]
Ciprofloxacin, 80%	DTS	Precipitation by mixing drug and polymer solutions	Stable at 25 °C/55% RH for 1 mo.	Improved dissolution and supersaturation	[67]
